# Adipokine and Hepatokines in Metabolic Dysfunction-Associated Steatotic Liver Disease (MASLD): Current and Developing Trends

**DOI:** 10.3390/biomedicines13081854

**Published:** 2025-07-30

**Authors:** Salvatore Pezzino, Stefano Puleo, Tonia Luca, Mariacarla Castorina, Sergio Castorina

**Affiliations:** 1 Department of Medicine and Surgery, University of Enna “Kore”, 94100 Enna, Italy; 2 Mediterranean Foundation “GB Morgagni”, 95125 Catania, Italytluca@unict.it (T.L.); sergio.castorina@unict.it (S.C.); 3 Department of Medical, Surgical Sciences and Advanced Technologies “G.F. Ingrassia”, University of Catania, 95123 Catania, Italy

**Keywords:** MASLD, adipokine, hepatokine, insulin resistance, oxidative stress, AMPK, fetuin, non-invasive biomarkers

## Abstract

**Background/Objectives:** Metabolic dysfunction-associated steatotic liver disease (MASLD) represents a major global health challenge characterized by complex adipose–liver interactions mediated by adipokines and hepatokines. Despite rapid field evolution, a comprehensive understanding of research trends and translational advances remains fragmented. This study systematically maps the scientific landscape through bibliometric analysis, identifying emerging domains and future clinical translation directions. **Methods:** A comprehensive bibliometric analysis of 1002 publications from 2004 to 2025 was performed using thematic mapping, temporal trend evaluation, and network analysis. Analysis included geographical and institutional distributions, thematic cluster identification, and research paradigm evolution assessment, focusing specifically on adipokine–hepatokine signaling mechanisms and clinical implications. **Results:** The United States and China are at the forefront of research output, whereas European institutions significantly contribute to mechanistic discoveries. The thematic map analysis reveals the motor/basic themes residing at the heart of the field, such as insulin resistance, fatty liver, metabolic syndrome, steatosis, fetuin-A, and other related factors that drive innovation. Basic clusters include metabolic foundations (obesity, adipose tissue, FGF21) and adipokine-centered subjects (adiponectin, leptin, NASH). New themes focus on inflammation, oxidative stress, gut microbiota, lipid metabolism, and hepatic stellate cells. Niche areas show targeted fronts such as exercise therapies, pediatric/novel adipokines (chemerin, vaspin, omentin-1), and advanced molecular processes that focus on AMPK and endoplasmic-reticulum stress. Temporal analysis shows a shift from single liver studies to whole models that include the gut microbiota, mitochondrial dysfunction, and interactions between other metabolic systems. The network analysis identifies nine major clusters: cardiovascular–metabolic links, adipokine–inflammatory pathways, hepatokine control, and new therapeutic domains such as microbiome interventions and cellular stress responses. **Conclusions:** In summary, this study delineates current trends and emerging areas within the field and elucidates connections between mechanistic research and clinical translation to provide guidance for future research and development in this rapidly evolving area.

## 1. Introduction

Previously known as nonalcoholic fatty liver disease (NAFLD), metabolic dysfunction-associated steatotic liver disease (MASLD) affects over one-third of the world population and is intimately connected with obesity, insulin resistance, and metabolic syndrome [1,2,3]. Because of its great frequency and potential for severe liver-related morbidity and death, MASLD presents major therapeutic problems ranging from simple hepatic steatosis to steatohepatitis, fibrosis, cirrhosis, and hepatocellular carcinoma [4,5]. With forecasts showing that MASLD prevalence will become the main cause of liver transplantation by 2030, epidemiological data suggest that MASLD prevalence has increased by 25% in the previous decade, therefore underlining the immediate need for efficient diagnosis and treatment plans [1,6,7]. Emerging as key mediators in the pathophysiology of MASLD, adipokines and hepatokines—secreted by adipose tissue and liver, respectively—have influenced insulin sensitivity, inflammation, and fibrogenesis [8,9]. Their dynamic interaction generates complicated signaling networks that control fibrotic development, oxidative stress responses, and hepatic fat accumulation [8,9,10,11]. Apart from important pathophysiological players, these protein mediators also show great potential as non-invasive indicators and therapeutic targets in MASLD treatment. Notwithstanding major progress in knowledge of MASLD pathophysiology, liver biopsy is still the gold standard for definite diagnosis and staging since it carries natural hazards of sample error, complications, and patient discomfort [12]. Particularly, the important transition to non-alcoholic steatohepatitis (NASH) and increasing fibrosis has strengthened research efforts to find dependable non-invasive biomarkers capable of precisely detecting various stages of MASLD [13]. Because of their mechanistic participation in disease progression and their detectability in circulation, adipokines and hepatokines have attracted a lot of interest in this respect [5,9,10,14,15].

Bibliometric analysis is the study of scholarly publications over a set period that uses numerous variables to obtain details regarding the state of the trends and subjects of a given field [16,17,18]. While prior bibliometric investigations have explored facets of MASLD pathogenesis, existing studies exhibit critical limitations in scope and methodology. Gu et al. (2023) focused closely on adipose tissue mechanisms in NAFLD, omitting hepatokines and the updated MASLD nomenclature [19]. Alam et al. (2024) analyzed gut microbiota interactions but did not address the integration of adipokine–hepatokine crosstalk [20], while Gong et al. (2025) restricted their investigation to pediatric populations, overlooking systemic metabolic drivers [21]. In contrast, our study represents the first comprehensive bibliometric analysis (2004–2025) to map the synergistic roles of adipokines and hepatokines in MASLD using multilayered methodologies. Unlike prior works relying on single tools (e.g., VOSviewer), we integrate Bibliometrix 5.0 and Biblioshiny 4.1. Different from the aforementioned studies, the objective of this work is to address the current trends in adipokine and hepatokine research in MASLD, highlight developing areas, approach uncovering latent connections between mechanistic research and clinical translation, address a critical gap in prior fragmented assessments, and offer fundamental advice for future research and development in this fast-expanding field.

## 2. Materials and Methods

Using the Scopus database—a reliable academic resource spanning millions of scholarly works—we conducted a thorough literature search [22,23]. To ensure comprehensive coverage up to the most recent literature available as of May, 2025, we applied a carefully constructed keyword search spanning publications from 2004 through that date. To minimize bias from ongoing database updates, all records were extracted and exported on the same day (19 May 2025). The search string used was as follows: (TITLE-ABS-KEY ((“adipokin*” OR “adipocytokin*” OR “hepatokin*”) AND (“nafld” OR “masld” OR “nonalcoholic fatty liver disease” OR “metabolic dysfunction-associated steatotic liver disease” OR “hepatic steatosis” OR “steatohepatitis”)) AND PUBYEAR > 2003 AND PUBYEAR < 2026 AND (LIMIT-TO (DOCTYPE, “ar”)) AND (LIMIT-TO (LANGUAGE, “English”))). The inclusion criteria were as follows: (1) peer-reviewed original articles in English; (2) publications from 2004 to 2025 until the date of extraction. The exclusion criteria included the following: (1) review articles; (2) duplicate publications; (3) non-English publications. Although the keywords were chosen with an eye toward generally accepted terminology, they guarantee comprehensive coverage of the field. By closely reading the obtained papers, we confirmed the success of our search approach. The obtained Scopus data was saved in CSV form. Different datasets can be handled and visualized using VOSviewer (version 1.6.20 [24]). We also used Bibliometrix and Biblioshiny tools for thematic mapping and trend analysis [25]. Installed and imported into R Studio (version 2024.04.2+764), Bibliometrix is an R package including several tools [25]. Using the R console, we started the Biblioshiny web-based utility meant to improve Bibliometrix package usage. Obtained in CSV form from Scopus, the bibliographic dataset was entered into the Biblioshiny interface for additional study [25].

## 3. Results

### 3.1. Publication Trends and Journal Contribution Patterns

**Figure 1:**
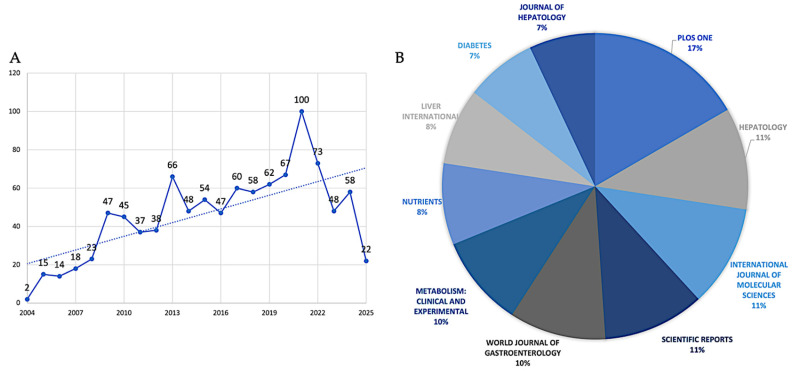
(**A**) temporal publication trends (2004–2025) and (**B**) top ten journal contributions (%) in the field.

Based on the parameters of the search, 1002 articles related to the searched field and covering the years 2004–2025 were retrieved from the Scopus database. The graph shows a time trend from 2004 to 2025 (Figure 1A), with actual values up to about 2022 and projections up to 2025 (dotted line). Starting from a minimum value of about two publications in 2004, there is an initially gradual growth. Around 2009, a significant acceleration point was observed with about 45–50 publications. After that, there is a steeper growth leading to a first local peak of about 65–70 publications around 2013. After 2016, there is a period of fluctuation, followed by a recovery that culminates in an absolute peak of around 100 publications in 2021. The final projection shows a trend that should be at 2021 levels.

**Figure 2:**
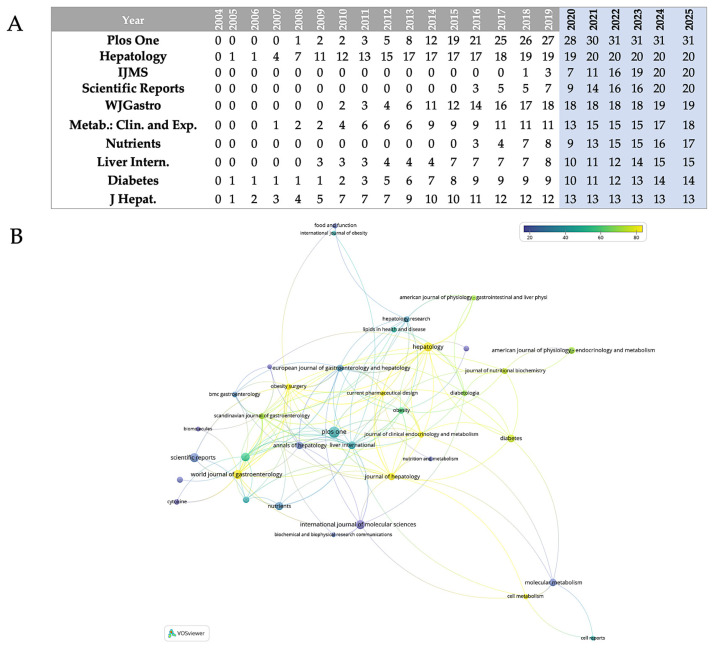
(**A**) Annual publications from the 10 most prolific journals in the field over about 20 years; IJMS = *International Journal of Molecular Sciences*; WJGastro = *World Journal of Gastroenterology*; Metab.: Clin. and Exp. = *Metabolism: Clinical and Experimental*; Liver Intern. = *Liver International*; J Hepat. = *Journal of Hepatology*. (**B**) Journal co-citation network generated via VOSviewer; nodes colored by citation impact (blue = low, yellow = high).

The relative contributions of the top ten journals that publish research in the field are quantified in Figure 1B. According to the data, there is a hierarchical structure among these top publications, with PLOS One standing out as the main contributor, with 17% of all publications. A secondary tier of productivity comprises three journals, which together account for 33% of publications: *Journal of Hepatology* (11%), *International Journal of Molecular Sciences* (11%), and *Scientific Reports* (11%). Comparing the third tier to the top four journals, the *World Journal of Gastroenterology* (10%) and *Metabolism: Clinical and Experimental* (10%) show a significant but somewhat lesser interest in the field. *Diabetes* (7%) and the *Journal of Hepatology* (7%) finish the ranking with the lowest portions among the top ten, while *Nutrients* (8%) and *Liver International* (8%) occupy the fourth tier. Different publication trajectories are revealed by the analysis of data (Figure 2A); the most striking growth pattern is seen in PLOS One, which began publishing in 2008 with just one paper in the field. By 2016, that number had risen to 19 papers, and by 2020, it had reached a peak of 31 annual publications. By maintaining this plateau of precisely 31 publications through 2025 (even if the analysis stops in May 2025), the journal has solidified its position as the leading publisher in this area. Hepatology exhibits earlier involvement, having published regularly since 2005 and continuing to produce a steady output of approximately 24 papers per year since 2013. This consistency is indicative of the journal’s well-established niche as a specialized hepatology publication. The most recent but steepest growth curve is shown by the *International Journal of Molecular Sciences*, which published its first article in 2018 (one paper) and then quickly expanded to 20 papers by 2025. Increased attention to the molecular mechanisms underlying MASLD is occurring at the same time as this acceleration. A similar late entry but quick growth is seen in *Scientific Reports*, which started with three papers in 2016 and reached 20 by 2024–2025, underscoring its increasing stature as a multidisciplinary platform. Consistent attention to the field is demonstrated by the *World Journal of Gastroenterology’s* steady growth from early involvement (two papers in 2010) to 19 papers annually by 2024–2025. The publication trajectories of the remaining journals are varied but generally increasing: *Metabolism: Clinical and Experimental* has grown from one paper in 2007 to seventeen by 2024; *Nutrients* has been delayed in entry (2016) but has grown rapidly to seventeen papers by 2024; *Liver International* has shown steady growth from three papers in 2009 to fifteen by 2025; and *Diabetes and Journal of Hepatology* have shown more moderate growth rates in recent years.

Figure 2B maps the co-citation interactions between journals in the field of adipokines and hepatokines in MASLD research. Journals are depicted as nodes in this representation, with connecting lines indicating bibliographic coupling or co-citation links between publications in these journals. The color gradient—blue (lower citation impact) to yellow (greater citation impact)—shows clearly which journals in this field of research have attracted more citations: yellow-green nodes symbolize the most cited publications with large citation counts. The network architecture shows several original journal clusters reflecting different subdisciplines that support the field research. Yellow–green higher-citation journals seem to prevail in the central-right area; they are most likely core articles in hepatology and metabolism that have set the foundation for the field’s fundamental literature. Blue-hued journals near the periphery most likely represent either more recent works or those from allied disciplines not as referenced in this particular field of research.

The citation analysis of significant articles in the field provides valuable insights into intellectual evolution and research objectives (Table 1).

In the most cited paper, Furukawa et al. [26] revealed that hypertrophic adipocytes in obesity overexpress NADPH oxidase while downregulating antioxidants like superoxide dismutase, generating systemic oxidative stress marked by elevated plasma TBARS and urinary 8-iso-PGF2α, inversely correlating with adiponectin levels. With approximately comparable citations in their respective studies, Petersen et al. [27] found that insulin resistance redirects postprandial energy from muscle glycogen synthesis to hepatic lipogenesis, explaining dyslipidemia independent of TNF-α/IL-6, while Lin et al. [28] showed that FGF21 requires adiponectin to mediate systemic metabolic effects, as adiponectin knockout mice resisted FGF21’s improvements in glucose tolerance and hepatic steatosis. To follow in the ranking, González-Périz et al. [29] demonstrated that omega-3 fatty acids resolve inflammation via specialized pro-resolving mediators, polarizing macrophages to anti-inflammatory phenotypes, and reducing hepatic steatosis in obese mice. Haukeland et al. [30] found that patients with NAFLD are characterized by a low-grade systemic inflammation, concluding that the high CCL2/MCP-1 levels in NASH might be of importance for the conversion from simple steatosis to NASH, and Bekri et al. [31] demonstrated hepcidin expression in both liver and adipose tissue, with elevated adipose mRNA in obesity correlating with inflammatory markers (IL-6, CRP), while IL-6 stimulated hepcidin in vitro; despite 68% of obese patients showing low transferrin saturation, adipose hepcidin lacked the iron-status feedback regulation observed in the liver, linking obesity-related inflammation to iron dysregulation [31]. In their paper, Ouchi et al. [32] characterized adiponectin’s dual role: full-length adiponectin activates hepatic AMPK to enhance fatty acid oxidation, while globular adiponectin suppresses TNF-α/NF-κB in Kupffer cells via cAMP/PKA. Subsequently, in terms of citation count, Smith et al. [33] linked adipose hypoxia in visceral fat to HIF-1α-driven lipolysis, hepatic insulin resistance, and de novo lipogenesis. In the penultimate position of the paper citation ranking, Jarrar et al. [34] identified elevated TNF-α and IL-8 in NAFLD patients versus controls, with strong inter-cytokine, and linked reduced adiponectin to insulin resistance; while visfatin was elevated in obesity, it declined in NASH, and TNF-α emerged as the sole independent predictor of fibrosis, with age, ALT, IL-8, and adiponectin distinguishing NASH from simple steatosis. Finally, Armstrong et al. [35] found that liraglutide induced NASH resolution in 39% of patients (vs. 9% placebo) and reduced fibrosis progression in the LEAN trial.

### 3.2. Geographic, Institutional, and Author Patterns

**Figure 3:**
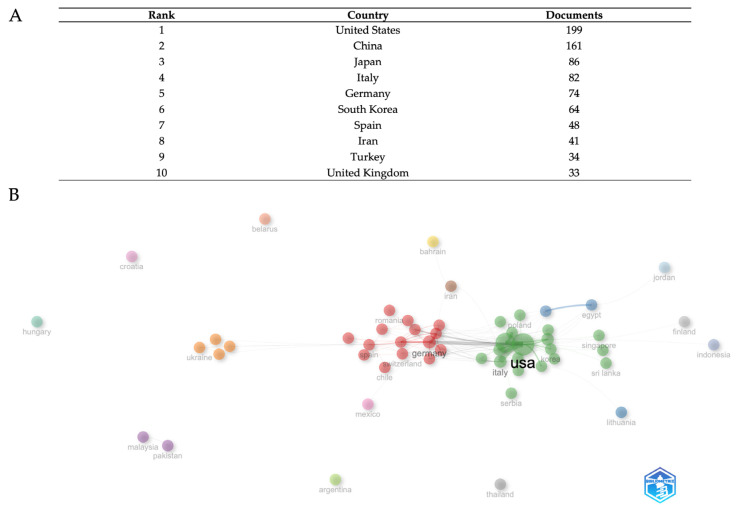
Geopolitical distribution of research output. The top ten countries for productivity in the field (**A**). Country-level collaboration network (**B**). The data were analyzed using Bibliometrix and Biblioshiny.

The bibliometric analysis of country field research reveals several trends in global production and collaboration (Figure 3A). With 190 documents, the United States comes top and clearly shows leadership in research output. With 161 papers, China is the second most productive nation; Japan ranks third with 86 papers. Ranked fourth and fifth, respectively, Italy and Germany have 82 and 74 documents, therefore reflecting significant European scientific effort. With 61 papers, South Korea ranks sixth and reflects its increasing importance in world research. Completing the top 10 are Spain, Iran, Turkey, and the United Kingdom; the respective document counts are 48, 41, 38, and 33. With a slow decrease in the quantity of documents among the other nations, this distribution shows a clear dominance of the United States and China, reflecting both regional strengths and the different scope of national research systems.

The network visualization (Figure 3B) illustrates several varied collaboration clusters, each denoted by different colors that indicate different clusters of international relationship intensity, and where the dimension of each node is proportional to country publication output. The United States and China serve as pivotal centers in the global collaboration network, maintaining broad ties with several countries across various regions. The positioning and clustering patterns indicate the influence of geographical closeness and thematic research alignments on collaboration strength. European nations seem to establish tight collaborative clusters, whereas Asian countries exhibit robust intra-regional relationships in addition to substantial trans-Pacific collaborations with North American institutions. The network structure demonstrates that research collaboration extends beyond basic bilateral interactions, creating intricate multinational research ecosystems that promote knowledge exchange and collaborative publication across various geographical and institutional barriers.

**Figure 4:**
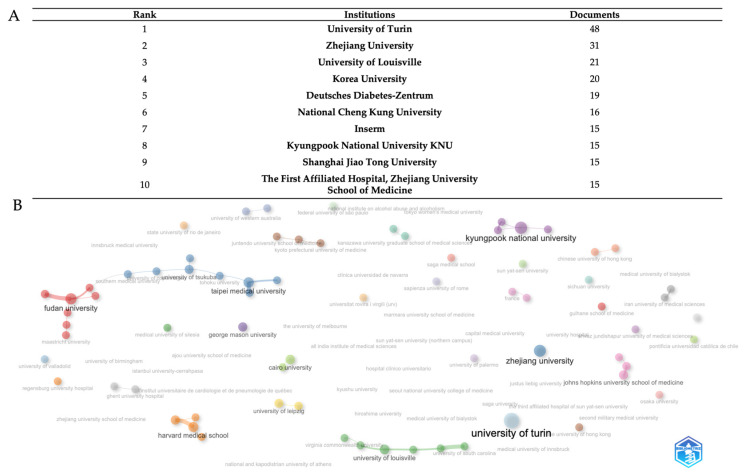
The top ten institutions for productivity in the field (**A**). Institution-level collaboration network (**B**). The data were analyzed using Bibliometrix and Biblioshiny.

At the institution level (Figure 4A), bibliometric analysis discloses a detailed ranking of the 10 leading research institutions, based on a total of 215 published documents from various international academic centers. The University of Turin exhibits dominant leadership with 48 documents, accounting for about 22% of total output, showcasing remarkable Italian research excellence and establishing a significant 15-document lead over the second-ranked school. Zhejiang University of China attains the second position with 31 publications (about 14%), signifying the most robust Asian academic representation in the elite echelon and illustrating China’s increasing investment in scientific research infrastructure. The University of Louisville ranks third with 21 documents (about 9.5%), showing the competitiveness of American institutions in this research subject.

The extensive institutional collaboration network of research institutes in the field is depicted in Figure 4B. Each concentric circle in the image represents a different academic or research organization, while the width of each node proportionately represents the volume of publications produced. The thickness of the connecting lines, which represent active collaborative links, indicates the frequency and level of inter-institutional cooperation. The visualization reveals a global institutional collaboration network comprising major research universities and medical centers organized into several distinct and main clusters representing regional and thematic research consortia in the field. The red cluster is dominated by Fudan University as the central hub, connected to Maastricht University and other Chinese institutions, representing the strongest Asian metabolic research network; the purple cluster with Kyungpook National University serves as the primary Asian hub with extensive connections to Korean and international partners, including the University Hospital and Juntendo University School of Medicine; the blue cluster with Zhejiang University and Taipei Medical University form a significant Sino-Taiwanese collaborative network, with connections extending to the Medical University of Innsbruck and other European institutions; the University of Turin (light-blue node) represents the European Mediterranean research hub, closely connected to the University of Louisville, the University of South Carolina, and multiple Italian medical centers, indicating strong transatlantic collaboration; in the orange clusters, Harvard Medical School and the University of Leipzig anchor this cluster with connections to Zhejiang University School of Medicine and German research institutions; Johns Hopkins University School of Medicine (pink cluster) maintains strategic connections across multiple networks. Cairo University (green) represents Middle Eastern participation. George Mason University (purple node) and Virginia Commonwealth University (green node) indicate specialized American research participation. This distributed network demonstrates mature international collaboration with strong regional hubs that maintain cross-continental partnerships, reflecting the global nature of metabolic disease research and the need for diverse patient populations and expertise in the field.

**Figure 5:**
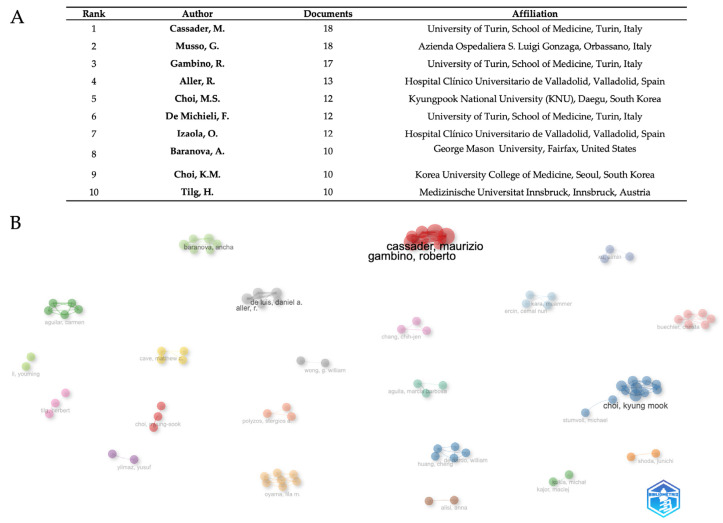
Author productivity (**A**) and networks (**B**). The data were analyzed using Bibliometrix and Biblioshiny.

Based on publication output, Figure 5A shows a complete ranking of the ten most prolific authors, therefore exposing important trends in academic research production and institutional affiliation distribution. With Cassader, M., and Musso, G. both reaching the highest productivity level of 18 documents, attesting to the pinnacle of scholarly output within the investigated research subject, the study shows a clear leadership dyad. Cassader, M. maintains affiliation with the University of Turin, School of Medicine, while Musso, G. operates from Azienda Ospedaliera S. Luigi Gonzaga in Orbassano, both institutions demonstrating exceptional Italian medical research excellence. With 17 papers, Gambino, R., places third, thus strengthening the University of Turin’s domination in the highest levels of the ranking. With 60% of the represented authors coming from European institutions and Italian institutions especially prominent through three academics placed in the top six rankings, the geographic distribution exposes European institutional supremacy. Two cooperative researchers from Hospital Clínico Universitario de Valladolid—Aller, R. (13 documents) and Izaola, O. (12 documents)—showcase Spanish representation exhibiting effective intra-institutional research collaborations. Asian contributions show up as 12 and 10 documents, respectively, from South Korean researchers Choi, M.S., from Kyungpook National University, and Choi, K.M., from Korea University College of Medicine. Baranova, A. from George Mason University (10 documents) shows North American academic presence; Austrian presence is shown by Tilg, H. from Medizinische Universität Innsbruck (10 documents). Establishing a mean output of 12.4 documents per author, the productivity distribution spans 18 documents maximum to 10 documents minimum, therefore demonstrating high standards for admittance into this top academic cohort. Four writers had the same production levels at the 10-document barrier, implying a natural competitive limit for top-tier academic recognition in the given study area. With regard to author-level cooperation networks (Figure 5B), the visualization exposes fifteen key unique clusters symbolizing specialized research communities in adipokine and hepatokine MASLD research. Representing an established Mediterranean metabolic research group, the dominating red cluster consists of Italian researchers Cassader, Maurizio, and Gambino, Roberto. Choi, Kyung Mook, leads the main blue cluster with Shumoff, Michael, signifying great Asian-international cooperation. Baranova, Ancha, and Aguilar, Carmen, and other members of the green cluster promote collaborations emphasizing genes and biomarkers. Other specialized clusters are the American cluster with Cave, Matthew C. (yellow), the Spanish group with De Luis, Daniel A., and Aller, R. (gray cluster), and specialist Asian groupings including Chang, Chih-jen (pink) and Shioda, Junichi (orange). Emerging clusters include Yilmaz, Yusuf (purple), Ponyzos, Stergios P. (salmon), Huang, Cheng with Reando, William (dark blue), Oyama, Lila M. (beige), Alisi, Anna (brown), and Gokto, Michal with Kajor, Maciej (light green). Li, Youming, Buechler, Christa, Kara, Muammer, Erich, Cemal Nuff, and Wong, G. William are further obvious researchers.

### 3.3. Research Theme Trends and Hotspots

#### 3.3.1. Analysis of Thematic Maps of Adipokine–Hepatokine MASLD Research

**Figure 6:**
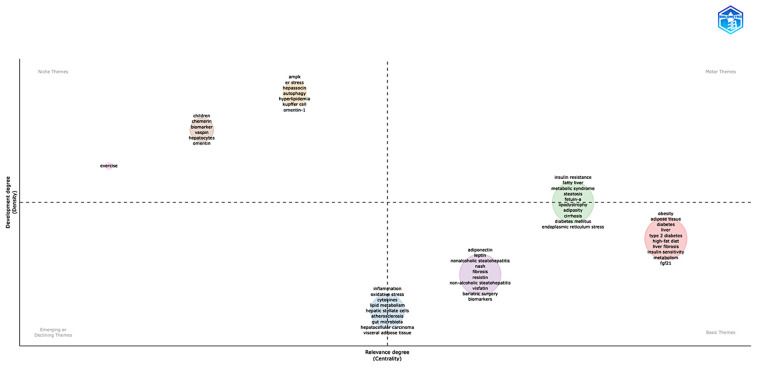
Thematic map analysis of research areas in the field. The horizontal axis (Centrality) measures the relevance of the theme in the general context, while the vertical axis (Density) indicates the level of development of the theme. The quadrants are divided into motor themes, basic themes, emerging or declining themes, and niche themes. The sizes of the circles represent the frequency or relative importance of each theme within the analyzed dataset. The data were analyzed using Bibliometrix and Biblioshiny.

Via the use of centrality–density dimensions, the thematic map analysis (Figure 6) reveals a sophisticated conceptual architecture arranged over four different quadrants. Representing very central and developed research areas that drive field innovation, motor/basic themes (green circle) comprise core pathophysiological concepts including insulin resistance, fatty liver, metabolic syndrome, steatosis, fetuin-A, lipo-dystrophy, adiposity, cirrhosis, diabetes mellitus, and endoplasmic reticulum stress. The red circle (right quadrant) covers metabolic foundations such as obesity, adipose tissue, diabetes, liver, type 2 diabetes, high-fat diet, liver fibrosis, insulin sensitivity, metabolism, and FGF21; the purple circle (left quadrant) contains adipokine-focused terms including adiponectin, leptin, nonalcoholic steatohepatitis (NASH), fibrosis, resistin, visfatin, bariatric surgery, and biomarkers. The light-blue circle basic/emerging themes span pathophysiological pathways including inflammation, oxidative stress, cytokines, lipid metabolism, hepatic stellate cells, atherosclerosis, gut microbiota, hepatocellular carcinoma, and visceral adipose tissue. Exercise interventions (pink circle), pediatric and novel adipokine research including children, chemerin, vaspin, hepatocytes, and omentin-1 (brown circle), and advanced molecular mechanisms encompassing AMPK, ER stress, hepassocin, hyperlipidemia, Kupffer cells, and omentin-1 (orange circle) show specialized focus areas through niche themes. The thematic map study exposes a sophisticated conceptual architecture arranged across centrality–density dimensions, defining research themes into four separate quadrants. Representing very central and developed research areas that drive field innovation, motor/basic themes (green circle) comprise core pathophysiological concepts including insulin resistance, fatty liver, metabolic syndrome, steatosis, fetuin-A, lipodystrophy, adiposity, cirrhosis, diabetes mellitus, and endoplasmic reticulum stress. In the basic theme quadrant, the red circle covers metabolic foundations such as obesity, adipose tissue, diabetes, liver, type 2 diabetes, high-fat diet, liver fibrosis, insulin sensitivity, metabolism, and FGF21. Also in the basic quadrant, the purple circle contains adipokine-focused terms, including adiponectin, leptin, nonalcoholic steatohepatitis (NASH), fibrosis, resistin, visfatin, bariatric surgery, and biomarkers. The light-blue circle basic/emerging themes span pathophysiological pathways including inflammation, oxidative stress, cytokines, lipid metabolism, hepatic stellate cells, atherosclerosis, gut microbiota, hepatocellular carcinoma, and visceral adipose tissue. In the niche theme, exercise interventions (pink circle), pediatric and novel adipokine research includes children, chemerin, vaspin, hepatocytes, and omentin (brown circle), and advanced molecular mechanisms encompassing AMPK, ER stress, hepassocin, hyperlipidemia, Kupffer cells, and omentin-1 (orange circle) show specialized focus areas.

#### 3.3.2. Temporal Trend Analysis of Research Themes in Adipokine–Hepatokine MASLD Investigation

**Figure 7:**
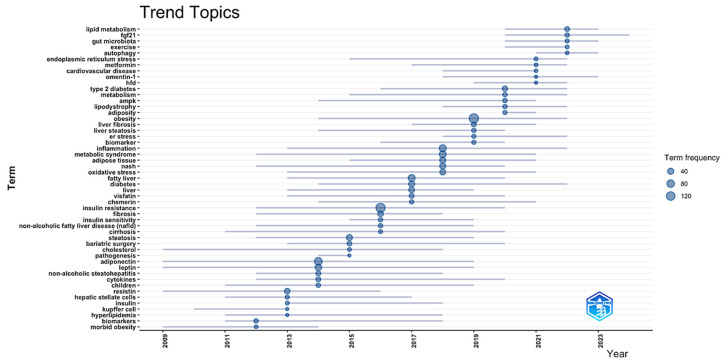
Temporal evolution of research topics (2014–2025). This sliding window analysis (3-year intervals) displays term emergence patterns using the thematic evolution function. Node size reflects normalized frequency within each period. Lines show the conceptual lineage between terms across periods. The data were analyzed using Bibliometrix and Biblioshiny.

From basic ideas to mechanistic specialization and clinical application, the temporal trend analysis (Figure 7) shows a complicated developmental path of study themes in adipokine–hepatokine MASLD investigations from 2009 to 2023, thus advancing the discipline. Reflecting the field’s main focus on basic disease characterization and demographic-specific investigations, the first research themes from 2009 to 2013 focused on vital pathophysiological concepts including “morbid obesity,” “biomarkers,” “hyperlipidemia,” “Kupffer cells,” “insulin,” “hepatic stellate cells,” and “children.” The period from 2013 to 2017 saw the emergence of fundamental metabolic and inflammatory concepts, including “cytokines,” “non-alcoholic steatohepatitis,” “leptin,” “adiponectin,” “pathogenesis,” “cholesterol,” “bariatric surgery,” “steatosis,” “cirrhosis,” “insulin sensitivity,” “fibrosis,” “insulin resistance,” “chemerin,” “visfatin,” “diabetes,” and “fatty liver,” indicating the field’s progression towards comprehending adipokine-mediated mechanisms and their clinical ramifications. The period from 2017 to 2023 illustrates a transition towards sophisticated molecular mechanisms and therapeutic targets, prominently including “oxidative stress,” “metabolic syndrome,” “inflammation,” “biomarker,” “endoplasmic reticulum stress,” “liver steatosis,” “liver fibrosis,” “obesity,” “adiposity,” “lipodystrophy,” “AMPK,” “metabolism,” “type 2 diabetes,” “skeletal muscle,” “cardiovascular disease,” “metformin,” “autophagy,” “exercise,” “gut microbiota,” and “lipid metabolism.” With larger circle sizes indicating greater word frequencies, particularly for newly developing mechanistic ideas like “autophagy,” “endoplasmic reticulum stress,” and “gut microbiota,” the temporal distribution pattern shows a rising study intensity in recent years. This points to the field’s direction toward integrated systems biology models, including inter-organ communication paths beyond traditional adipokine–hepatokine paradigms. This chronological development shows how the field evolved from descriptive clinical studies to mechanistic research, including cellular stress responses, metabolic control, and therapeutic intervention strategies, highlighting the growing recognition of MASLD as a complex systemic disease needing multitarget therapeutic approaches to address both adipose tissue dysfunction and hepatic pathology.

Table 2 summarizes the key thematic shifts identified across the full 2004–2025 window, listing for each period the dominant keywords, pivotal discoveries, and the most influential references.

#### 3.3.3. Network Analysis of MASLD’s Adipokine–Hepatokine Research Clusters: Updated

**Figure 8:**
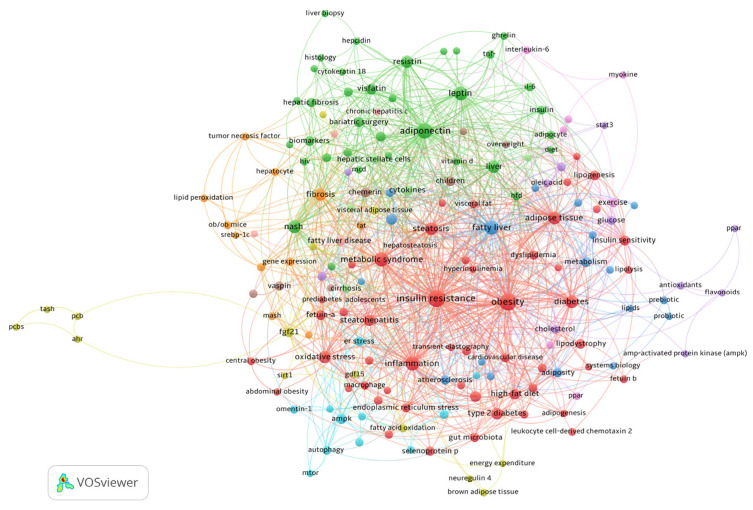
Clustered co-occurrence map extracted from the keywords of 1002 publications. In the map, the sizes of the frames indicate the frequency with which the keyword occurred. The co-occurrence strength between pairs of keywords is indicated by the proximity of two nodes and the thickness of the line connecting them. The color of the circle denotes keyword clusters, which are usually composed of co-occurring terms and can be regarded as broad research subjects in the field.

Several thematic clusters in the field were identified through author keyword co-occurrence analysis (Figure 8). In the network representation, the node size indicates the frequency of the keyword. The closeness of two nodes and the line’s thickness linking them signify the co-occurrence strength between keyword pairs. The color of the nodes signifies keyword clusters, which often encompass co-occurring terms and can be understood as overarching study themes in the discipline.

The cluster colored in red combines several terms relative to cardiovascular comorbidities, such as visceral fat, insulin resistance, type-2 diabetes, abdominal obesity, and cardiovascular disease, with metabolic dysfunction and endocrine disorders associated with selenoprotein P, fetuin-A, and fetuin-B. This cluster demonstrates the connection between systemic metabolic dysregulation and non-invasive monitoring techniques by linking metabolic processes, such as lipogenesis and lipolysis, with diagnostic techniques like transient elastography.

Adipokine pathways and inflammatory markers like adiponectin, leptin, and IL-6 are the focus of the green cluster, which is closely related to histological evaluations of cirrhosis and hepatic fibrosis. This cluster’s translational focus is highlighted by clinical treatments like bariatric surgery and biomarkers like cytokeratin 18, which connect advanced liver pathology to dysfunctional adipose tissue.

Focusing on progressive liver injury, the orange cluster emphasizes fibrotic processes fueled by hepatocyte damage, lipid peroxidation, and TNF-α. *Ob*/*ob* mice are one type of experimental model that provides mechanistic insights into the pathophysiology of MASH. The molecular mechanisms underlying extracellular matrix remodeling are highlighted by advanced gene expression studies in this cluster.

By examining metabolic homeostasis through hepatokines like FGF21 and GDF15, the yellow cluster establishes links between energy expenditure, fatty acid oxidation, and brown adipose tissue activity. This cluster finds new targets for treatment that affect systemic metabolism.

The brown cluster, which studies early metabolic abnormalities in children and overweight people, is characterized by specialized terms like chemerin and vaspin, which shed light on the developmental paths of MASLD.

The pink cluster provides an example of how physical activity affects metabolic modulation. Here, exercise-induced myokines like interleukin-6 interact with PPAR pathways to affect hepatic lipid metabolism. The AMPK and PPAR pathways are important targets for addressing oxidative stress and cholesterol homeostasis.

The purple cluster contains terms related to how antioxidants, flavonoids, and STAT3 signaling regulate lipid metabolism.

The blue cluster presents terms on the prebiotic and probiotic terms, adiposity, cholesterol, and fatty liver.

Finally, the light-blue cluster contains terms relating to the cellular stress responses, including mTOR signaling and ER stress, while emphasizing protective factors, such as omentin-1, that could offer treatment alternatives to lessen hepatocyte damage.

## 4. Discussion

Our bibliometric study includes 1002 papers on MASLD that were published between 2004 and May 2025. This is the largest dataset that was available at the time of extraction. Figure 1A shows an S-shaped growth path that starts with a period of incubation from 2004 to 2009, then quickly turns around and ends with about 70 publications by 2013. By 2021, the number of papers published each year will have leveled off at around 100. This path shows how the discipline has moved from descriptive phenotyping to mechanistic precision, and then, since 2022, it has been strategically consolidating with specialized specialization and precision medicine applications. The journal landscape (Figure 1B) highlights methodological diversity: PLOS ONE comprises 17% of records, dominating open-access contributions in this field, while leading hepatology journals (*Journal of Hepatology, Hepatology, Liver International*) serve as the foundation of clinical scholarship. Multidisciplinary journals like *Scientific Reports*, the *International Journal of Molecular Sciences, Diabetes, Metabolism: Clinical and Experimental, Nutrients*, and the *World Journal of Gastroenterology* bring together molecular, endocrine, nutritional, and gastroenterological frameworks in discussions about hepatology. These patterns show how the field has changed over time and how it is now focused on turning mechanistic findings into MASLD treatments that are very specific to each patient.

Annual publication trends for the top ten most productive journals in terms of publication count indicate specific developmental trajectories (Figure 2A). With core hepatology journals (*Hepatology, Journal of Hepatology*) forming a strongly interconnected nucleus, the journal co-citation network (Figure 2B) displays a multidisciplinary structure with PLOS ONE serving as the main hub connecting thematic clusters. The interdisciplinary scope and co-citation patterns of the field are reflected in the strategic bridge positions held by multidisciplinary outlets such as *Scientific Reports*, *International Journal of Molecular Sciences*, and *World Journal of Gastroenterology*, and the integration of nutritional and metabolic perspectives by specialized endocrinology journals like *Diabetes*.

The bibliometric citation analysis of the most cited papers in the field (Table 1) reveals foundational insights into disease mechanisms and therapeutic targets. These studies collectively map NAFLD’s progression from adipose-liver crosstalk to fibrosis, emphasizing oxidative stress, adipocytokine imbalance, and multi-organ metabolic dysfunction as therapeutic priorities. The citation trends reflect evolving paradigms from the “two-hit hypothesis” to precision medicine approaches targeting genetic, inflammatory, and metabolic drivers.

The MASLD/NAFLD research scenario in the field is divided into geographical and thematic poles (Figure 3), with the United States (199 publications) and China (161) dominating, together with 36% of global production, while emerging countries such as Iran and Turkey show growth but little international integration. Europe, led by Italy (82) and Germany (74), maintains transatlantic collaborations focused on translational studies.

At the institutional level (Figure 4A,B), the University of Turin (48) emerges as a leader, followed by Zhejiang (31), with collaborative networks organized in regional clusters: Asian (Fudan/Maastricht), Euro-American (Turin/Louisville), and Korean (Kyungpook). This regionalization, while favoring specialization, risks fragmenting the holistic understanding of the disease, especially in underrepresented populations in the Middle East and Latin America, where the burden is greatest. Author-centric networks (Figure 5B) reflect consolidated expertise but little transdisciplinary interconnection. The future challenge lies in creating inclusive collaborative platforms, standardizing protocols, and aligning research priorities with the needs of endemic areas, integrating genomic, clinical, and socio-environmental approaches for equitable and effective therapeutic strategies.

An examination of thematic maps in adipokine–hepatokine research within MASLD (Figure 6) delineates a stratified conceptual framework with distinct clusters positioned across centrality–density dimensions that define their thematic classification and research maturity. The green cluster functions as a basic theme/motor theme that underpins fundamental pathogenic mechanisms, with Fetuin-A identified as a potential diagnostic hepatokine exhibiting elevated serum concentrations in MASLD patients compared to controls, correlating with ultrasound steatosis grades and insulin resistance through AMPK inhibition [36,37,38,39,40,41,42]. The purple cluster represents a basic theme, emphasizing mature adipokine networks wherein bariatric surgery reinstates the adiponectin/leptin equilibrium [43,44,45], and combinatorial biomarkers (adiponectin/leptin ratio + HOMA-IR) attain enhanced diagnostic precision for predicting metabolic syndrome [46]. Red cluster investigations, also positioned as a basic theme, designate FGF21 as a crucial hepatokine, exhibiting hepatic mRNA expression that is 9 times elevated in MASLD patients, while phase 2 trials reveal that pegozafermin analogs diminish hepatic fat by 45% through PPARα activation [47,48]. The orange cluster, representing a niche theme, illustrates sophisticated molecular mechanisms, highlighting the regulation of macrophage polarization by Kupffer cell-derived miR-690 and the modulation of TNF-α/ER stress pathways by omentin-1, which significantly reduces inflammatory markers without affecting hepatic steatosis [49,50,51]. Specialized themes also encompass the pink cluster as a niche theme regarding exercise-induced myokines such as neuregulin-4 (Nrg4), which mitigates steatosis via Nrg4/ErbB4/AKT signaling and demonstrates rapid effects on hepatic lipid metabolic reprogramming following sleeve gastrectomy [52,53]. The brown cluster, also representing a niche theme, associates pediatric MASLD with chemerin’s dual inflammatory and anti-inflammatory functions, though it is notably showing elevated levels in NAFL patients, but no significant differences between NASH patients and controls [54]. The light-blue cluster appears positioned as an emerging or declining theme, connecting mechanistic insights via inflammation, oxidative stress, and interactions with gut microbiota, substantiated by evidence that specialized pro-resolving mediators from omega-3 fatty acids effectively mitigate hepatic inflammation through macrophage polarization [55,56].

Temporal trend analysis (Figure 7) reveals a complicated change in research goals reflecting the field’s progress from descriptive pathophysiology to mechanistic precision and therapeutic translation. Fundamental ideas, including morbid obesity, biomarkers, and hepatic stellate cells, defined the first phase (2009–2013), therefore reflecting the early recognition of adipose–liver connections and fibrogenic processes. With Kupffer cells emphasizing first investigations on hepatic macrophage polarization and the inflammatory pathways allowing the advancement of steatosis, this period set the groundwork for understanding NAFLD as a multisystem illness [57,58,59]. Adipokine signaling networks—especially leptin, adiponectin, chemerin, and visfatin—emerged in concert with basic pathophysiological processes, including insulin resistance, fibrosis, and bariatric surgery as a therapeutic intervention during the middle period (2013–2017) [44,60]. While the dual role of leptin in metabolic control and fibrogenesis has become known, this temporal clustering corresponds with important studies that show adiponectin’s hepatoprotective effects via AMPK activation and its inverse relationship with NASH severity [61,62,63]. Supported by new meta-analyses confirming its rise in MASLD patients and its link with the degree of hepatic steatosis [54], the concomitant development of chemerin implies an expanding awareness of new adipokines. With an eye toward endoplasmic reticulum stress, autophagy, gut flora, and AMPK signaling, the modern period (2017–2023) shows a notable turn toward a systems-level mechanistic knowledge. This development supports the results of our thematic study, in which these ideas occupied important thematic positions, indicating their development from new frontiers to accepted research drivers. In modern research, FGF21’s importance matches clinical validation studies showing its upregulation in MASH patients and the therapeutic potential of FGF21 analogs in enabling both MASH resolution and fibrosis improvement [47,64,65,66,67,68,69,70,71]. With reduced autophagic function seen in aging and high-fat diet models that predispose individuals to NASH [72], the simultaneous appearance of autophagy underlines its significance as a regulating mechanism for lipid droplet destruction and hepatocyte survival. Especially noteworthy is the late introduction of gut microbiota (post-2020), which contrasts with its prominent importance in our conceptual framework analysis and shows a quick absorption of microbiome science into mainstream MASLD research. Recent studies show that gut dysbiosis—more especially, an enhanced *Firmicutes*/*Bacteroidetes* ratio—helps to cause hepatic steatosis using increased lipopolysaccharide translocation and TLR4-mediated inflammatory pathways [73,74]. The recent temporal convergence of lipid metabolism, exercise, and cardiovascular disease emphasizes the growing awareness of MASLD as a systemic metabolic disease requiring multitarget therapy approaches. Clinical studies showing the synergistic impact of lifestyle changes along with pharmaceutical modulators of hepatokine networks confirm this [64,75].

The network analysis (Figure 8) demonstrates a multifaceted scenario in the field. A deeper examination of the main terms within the distinct color-coded clusters reveals different thematic domains of research to which each cluster focuses.

The red cluster encompasses a range of terms that together characterize MASLD’s terrain of systematic metabolic dysfunction. Metabolic syndrome, obesity, and insulin resistance—all of which are closely related to cardiovascular comorbidities, including atherosclerosis—are major members of this cluster. Within this paradigm, selenoprotein P and fetuin-A and B become essential mediators since they worsen hepatic lipotoxicity via modulating insulin signaling pathways [64,76,77,78,79]. Especially observed at high levels in MASLD, fetuin-B aggravates insulin resistance by blocking AMPK and activating the LXR-SREBP1c axis, hence fostering hepatic lipogenesis [80,81]. Serum fetuin-B has been shown in clinical investigations to be favorably correlated with indicators of adipose tissue inflammation [82,83], including IL-6 and TNF-α, as well as the degree of hepatic steatosis [84]. Furthermore, fetuin-B establishes a metabolic feedback loop in adipocytes by interacting with the insulin receptor-β, therefore sustaining the interaction between hepatic steatosis and peripheral insulin resistance [85]. Further evidence of fetuin-B’s participation in MASLD etiopathogenesis is provided by the discovery that dietary therapeutic interventions lowering fetuin-B levels by about 9.5% have been observed to increase insulin sensitivity, therefore underscoring its possible use as a therapy target [84]. To underline their significance in disease development, the cluster also includes terms connected to the gut microbiota and visceral obesity [86,87]. While visceral obesity induces hepatic inflammation via the release of macrophage-derived cytokines such as IL-1β and MCP-1, the dysbiosis of the gut microbiota increases the absorption of saturated fatty acids [88,89]. The terms in the red cluster taken together show the intricate, multifactorial network of metabolic, inflammatory, and endocrine elements supporting the pathophysiology and clinical manifestations of MASLD.

The green cluster highlights the central role of inflammatory pathways and adipokine dysregulation in the progression of MASLD. Key terms such as IL-6 and TNF-α underscore the importance of chronic inflammation, as these cytokines drive hepatic inflammation, insulin resistance, and fibrogenesis by activating hepatic stellate cells and promoting a pro-fibrotic microenvironment [90]. The green cluster also includes terms related to clinical interventions and non-invasive biomarkers. Bariatric surgery decreases hepatic fibrosis through the normalization of ghrelin and hepcidin levels [45,91]. Non-invasive biomarkers such as cytokeratin-18 [92,93,94,95] and vitamin D function as non-invasive biomarkers [96,97,98]. The significant correlation of resistin with fibrosis contrasts with the protective function of adiponectin, highlighting the differing roles of adipokines [99,100,101,102,103,104,105].

The orange cluster underscores the critical significance of oxidative stress in promoting fibrogenesis in MASLD. Key terms in this cluster, including TNF-α, indicate its role in facilitating hepatic stellate cell activation via the miR-93/SIRT1 signaling pathway, especially in *ob*/*ob* models of metabolic dysfunction [106]. In this situation, TNF-α elevates oxidative stress and upregulates miR-93, inhibiting SIRT1, a crucial regulator of antioxidant defenses and anti-fibrotic activities [106]. The resultant imbalance expedites collagen accumulation and fibrosis. The findings highlight the significance of targeting oxidative stress pathways, such as reinstating SIRT1 activity or decreasing TNF-α, as prospective therapeutic approaches to alleviating fibrogenic development in MASLD.

The yellow cluster identifies FGF21 as hepatoprotective, countering fetuin-A-induced insulin resistance [64,66,71,76,77], while NRG4 connects exercise to lipid oxidation [52,107]. Regarding the identified term (GDF15 Growth/differentiation factor 15), it functions as both a dual biomarker and a therapeutic target in MASLD, with serum concentrations correlating with the severity of hepatic steatosis and predicting the risk of liver cancer in individuals exhibiting a Fib-4 index greater than 1.3-fold [108,109,110]. Mechanistically, hepatocyte-derived GDF15 engages with FGF21 to promote fatty acid oxidation through PPARα activation while inhibiting VLDL formation; yet, its overexpression paradoxically elevates ER stress indicators under normolipidemic circumstances [111,112].

The brown cluster emphasizes the functions of chemerin and vaspin in MASLD. Chemerin is linked to heightened inflammation and insulin resistance, positioning it as a possible marker of disease severity [91,113,114]. Conversely, vaspin exhibits anti-steatotic properties in laboratory models; however, the clinical results are conflicting, primarily due to the diversity in assay methodologies [115,116]. This highlights the necessity of consistent measuring techniques to elucidate the clinical value of these adipokines as biomarkers or therapeutic targets in MASLD.

The pink cluster emphasizes the advantages of exercise and the regulating roles of myokines and nuclear receptors in MASLD. Important terms in this cluster are myokines and nuclear receptors like PPAR, which coordinate metabolic changes in response to physical activity [107,117]. Exercise stimulates myokines—mostly NRG4—which have protective effects on hepatic metabolism to be released [118,119]. Regarding the term IL-6, it has two functions: it upregulates the expression of fatty acid-binding proteins such as L-FABP, thus facilitating effective post-prandial lipid transfer [120], and it activates PPARα in the liver, thus boosting fatty acid oxidation [121]. Exercise-induced IL-6 simultaneously reduces hepatic PPARγ expression, hence lowering de novo lipogenesis [120]. Through encouraging lipolysis and thermogenesis in adipose tissue, this change not only reduces hepatic fat storage but also improves general metabolic health [120,122]. Since physical activity directly alters important controllers of lipid metabolism and energy expenditure, the coordinated action of these pathways emphasizes the therapeutic possibilities of physical activity in MASLD [120,122].

The purple cluster includes therapeutic agents like flavonoids and statins, emphasizing their functions in metabolic regulation and liver protection. Statins, such as rosuvastatin, activate AMPK through LKB1 phosphorylation, resulting in mTORC1 inhibition and a notable decrease in hepatic fat accumulation, up to 30%, by inhibiting SREBP-1c-mediated lipogenesis [123]. Antioxidants, especially flavonoids, have shown effectiveness in enhancing outcomes in obesity and non-alcoholic fatty liver disease (NAFLD) [16]. The citrus flavonoid nobiletin enhances insulin sensitivity and decreases hepatic steatosis without AMPK activation, instead influencing the LXR and SREBP-1c pathways to suppress hepatic lipogenesis [124]. The findings highlight the therapeutic potential of targeting metabolic and oxidative stress pathways in MASLD.

The blue cluster concentrated on prebiotics/probiotics, obesity, cholesterol, and hepatic steatosis. Studies found that *Lactobacillus plantarum* AR113 diminishes hepatic triglycerides by 35% via the AMPK-mediated suppression of SREBP-1c pathways [125,126], whereas synbiotics decrease secondary bile acids associated with the advancement of steatosis [127,128,129]. Moreover, it has been shown that inulin supplementation elevates the *Akkermansia muciniphila* abundance fivefold, improving intestinal barrier integrity and diminishing TLR4-mediated hepatic inflammation in NAFLD mice [127,128,129].

The light-blue cluster includes essential cellular stress terms related to processes such as mTOR signaling and ER stress pathways in the pathophysiology of MASLD. The dysregulation of mTOR promotes hepatic steatosis through SREBP-mediated lipogenesis, while liver-specific PPDPF deletion induces spontaneous fatty liver by negatively regulating the mTORC1-S6K-SREBP1 signaling pathway [130]. In contrast, specific hepatic mTORC1 suppression via folliculin (FLCN) deletion safeguards against NAFLD/NASH by promoting TFE3-mediated lipid clearance and inhibiting de novo lipogenesis through TFE3-induced Insig2 expression [131]. On the other hand, chronic endoplasmic reticulum stress exacerbates NAFLD progression by stimulating lipogenesis, restricting very-low-density lipoprotein production, and enhancing insulin resistance via unfolded protein response pathway activation, with PERK-mediated Nrf2 phosphorylation offering antioxidant defense [132]. Within this context, omentin-1 functions as a protective adipokine, regulating AMPKα/mTOR signaling and maintaining autophagy in mice subjected to a high-fat diet, while AMPK inhibition negates these advantageous effects [133]. Collectively, the light-blue cluster underscores the importance of targeting cellular stress and metabolic signaling pathways to mitigate MASLD progression.

This bibliometric study acknowledges methodological considerations inherent to the approach. Our exclusive reliance on Scopus, while ensuring reproducibility, may introduce geographical and linguistic preferences that could underrepresent non-English research contributions. The quantitative nature excels at revealing research patterns and networks, though it focuses on citation metrics rather than individual study quality. Temporal projections to 2025 utilize validated statistical modeling, though future directions may be influenced by emerging clinical priorities not fully captured in current data. Despite these characteristics of bibliometric methodology, our analysis provides valuable insights into the evolving MASLD-organokine research landscape.

## 5. Conclusions

From descriptive studies of pathophysiology, MASLD research has developed into integrated models addressing the complexities of systemic metabolic dysregulation. Recent advances underline the validation of non-invasive biomarkers such as fetuin-A and FGF21, which have shown diagnostic and prognostic significance for MASLD and are progressively being included in research and clinical assessment systems. The field has also witnessed the emergence of novel therapeutic strategies targeting the adipokine-hepatokine axis, with mechanistic studies elucidating the roles of key mediators like adiponectin, leptin, and FGF21 in hepatic steatosis, inflammation, and fibrosis. Thematic studies show a change toward knowledge of the interactions among adipose tissue, liver, and other organs, including the gut–liver axis, mitochondrial activity, and inter-organ communication networks. Reflecting the growing emphasis on systems biology and precision medicine methods, advanced molecular research currently investigates oxidative stress, autophagy, endoplasmic reticulum stress, and the involvement of myokines and pediatric-specific adipokine profiles. This development highlights a growing agreement among experts that MASLD is a multisystem disease needing multimodal treatments and is inspiring translational research, combinatorial therapeutics, and biomarker discoveries to address early diagnosis and efficient treatment needs.

## Figures and Tables

**Table 1:** Top 10 Cited Papers (2004–2025). TC = total citations. The data were analyzed using Bibliometrix and Biblioshiny.

Paper	TC	TC per Year	Ref.
Furukawa S, 2004, J Clin Invest	4560	207.27	[26]
Petersen Kf, 2007, Proc Natl Acad Sci USA	600	31.58	[27]
Lin Z, 2013, Cell Metab	592	45.54	[28]
González-Périz A, 2009, Faseb J	503	29.59	[29]
Haukeland Jw, 2006, J Hepatol	502	25.10	[30]
Bekri S, 2006, Gastroenterology	418	20.90	[31]
Ouchi N, 2010, Science	416	26.00	[32]
Smith U, 2016, J Intern Med (Gbr)	389	38.90	[33]
Jarrar Mh, 2008, Aliment Pharmacol Ther	380	21.11	[34]
Armstrong Mj, 2016, J Hepatol	367	36.70	[35]

**Table 2:** Key thematic shifts identified in MASLD research (2004–2025).

Period (Years)	Key Research Focus	Dominant Themes	Research Paradigm	Clinical Translation
**2009–2013** **(Foundation Phase)**	Basic disease characterization Initial NAFLD recognition Oxidative stress foundations Morbid obesity Basic biomarkers Kupffer cells Hepatic stellate cells Pediatric aspects	Adipose-liver crosstalk Two-hit hypothesis Inflammatory markers Basic pathophysiology Hyperlipidemia Insulin dysfunction Children-specific research Cellular stress responses	Descriptive Phenotyping & Basic Disease Characterization	Limited clinical application & Biomarker identification
**2013–2017** **(Mechanistic Phase)**	Adipokine signaling networks Leptin and adiponectin mechanisms Insulin resistance pathways Bariatric surgery effects Chemerin and visfatin emergence	Cytokines (IL-6, TNF-α) NASH progression Fibrosis development Metabolic syndrome Cholesterol metabolism Pathogenesis mechanisms	Mechanistic Understanding	Therapeutic intervention strategies
**2017–2023** **(Systems Biology Phase)**	Molecular mechanisms Endoplasmic reticulum stress Autophagy Gut microbiota integration AMPK signaling Exercise-induced myokines	Oxidative stress Metabolic reprogramming Inter-organ communication Gut-liver axis Cardiovascular comorbidities Metformin therapy Lipid metabolism	Systems Biology Integration	Multi-target therapeutic approaches
**2021–2025** **(Consolidation Phase)**	Precision medicine applications Non-invasive biomarker panels Therapeutic target validation Clinical translation focus	Integrated systems models Fetuin-A as diagnostic marker FGF21 therapeutic potential Precision biomarkers Multimodal treatments	Precision Medicine Application	Clinical validation & implementation

## Data Availability

The original contributions presented in this study are included in the article. Further inquiries can be directed to the corresponding author.

## Abbreviations

The following abbreviations are used in this manuscript:

AMPK	AMP-activated protein kinase
CSV	Comma-separated values
ER	Endoplasmic reticulum
FGF21	Fibroblast growth factor 21
Fib-4	Fibrosis-4 Index
GDF15	Growth differentiation factor 15
HOMA-IR	Homeostatic Model Assessment for Insulin Resistance
IL-6	Interleukin-6
Insig2	Insulin Induced Gene 2
L-FABP	Liver Fatty Acid-Binding Protein
LXR	Liver X Receptor
MASLD	Metabolic dysfunction-associated steatotic liver disease
MCP-1	Monocyte chemoattractant protein-1
mTOR	Mechanistic target of rapamycin
mTORC1	mammalian target of rapamycin complex 1
NAFLD	Nonalcoholic fatty liver disease
NASH	Nonalcoholic steatohepatitis
Nrf2	Nuclear Factor Erythroid 2-related factor 2
NRG4	Neuregulin-4
PERK	protein kinase RNA-like endoplasmic reticulum
PPAR	Peroxisome proliferator-activated receptor
PPDPF	Pancreatic Progenitor Cell Differentiation and Proliferation Factor)
S6K	Ribosomal S6 Kinase
SREBP-1c	Sterol regulatory element-binding protein 1c
TFE3	Transcription factor E3
TLR4	Toll-like receptor 4
TNF-α	Tumor necrosis factor-alpha
VLDL	Very-low-density lipoprotein
